# Molecular signatures of selective motor neuron vulnerability in old age

**DOI:** 10.1113/EP094279

**Published:** 2026-10-21

**Authors:** Sang Won Cheung, Matthew J. Fogarty

**Affiliations:** ^1^ Department of Physiology & Biomedical Engineering Mayo Clinic Rochester Minnesota USA; ^2^ Department of Neurology Mayo Clinic Rochester Minnesota USA

**Keywords:** brainstem, Chodl, hypoglossal, lumbar, MMP‐9, spinal cord

## Abstract

Motor neuron (MN) death and impairment of fast‐fatigable motor units is widely recognized in elderly humans and rats. However, the molecular signatures identifying vulnerable MNs remains underexplored. In murine models of amyotrophic lateral sclerosis, matrix metalloproteinase 9 (MMP‐9) and chondrolectin (Chodl) decline with disease progression and have been considered molecular markers of fast‐fatigable MNs. However, their expression in brainstem and lumbar MNs, in accordance with the ‘size principle’ (in which motor units are recruited in a size‐dependent manner, with MNs of lower capacitance activated before those of greater capacitance) and their expression throughout the lifespan remain unclear. Here, we evaluated MMP‐9 and Chodl in lumbar and hypoglossal MNs in young (6 months) and old (24 months) Fischer 344 (F344) rats. Longitudinal lumbar spinal cord transverse brainstem sections (the orientations providing maximal MN somal sizes for each pool) were immunolabelled for choline acetyltransferase, MMP‐9 and Chodl. Expression was quantified using confocal microscopy in young and old rats (*n* = 5 females, *n* = 5 males per age). Overall, MMP‐9 demonstrated a positive association with lumbar MN somal surface area in young rats. Paired within‐animal analysis showed that in larger MNs, MMP‐9 positivity was ∼70% greater than smaller MNs. The size‐dependence of MMP‐9 expression was absent in old lumbar MNs, suggesting either selective loss or downregulation of MMP‐9 MN expression in ageing. No size‐dependent change in Chodl was observed in lumbar or hypoglossal MNs, nor was MMP‐9 associated with MN size in hypoglossal MNs. Collectively, these findings identify MMP‐9 as a marker enriched within large lumbar MNs in young rats and suggest that age‐related alterations in MMP‐9 expression may reflect the selective vulnerability of the lumbar MNs.

## Abstract (highlights)

- **What is the central question of this study?**
  Matrix metallopeptidase‐9 (MMP‐9) and chondrolectin (Chodl) are considered markers of vulnerable fast motor neurons (MNs) in amyotrophic lateral sclerosis models: how do they adhere to the ‘size principle’ and what is their age‐related loss in rat hypoglossal and lumbar MNs?
- **What is the main finding and its importance?**
  MMP‐9 was predominantly expressed in larger young rat lumbar MNs, but this pattern was not observed in old lumbar MNs or in hypoglossal MNs of any age, or with Chodl. These findings are consistent with fast MN death or reduced MMP‐9 expression in old lumbar MNs.

## 1 INTRODUCTION

Motor neurons (MNs) in the brainstem and spinal cord are central to all voluntary and reflex movements. The motor unit (an individual MN and the striated muscle fibres it innervates) is the key functional element of neuromotor control, with motor unit type heterogeneity underlying the matching of endurance and power requirements to desired behaviours (Kernell, 1986). Slow and fast fatigue resistant motor units (type S and FR) comprise smaller MNs innervating low force, high‐endurance type I and IIa muscle fibres, respectively (Fogarty & Sieck, 2019). Fast fatigable motor units (type FF) comprise larger MNs innervating higher‐force, highly fatigable type IIx/b muscle fibres (Fogarty & Sieck, 2019). Motor units are recruited in a size‐dependent manner, with MNs of lower capacitance activated before those of greater capacitance (i.e., type S before FR before FF), a phenomenon known as the ‘size principle’ (Henneman, 1957; Zajac & Faden, 1985). While the myosin heavy chain (MyHC) molecular signatures of striated muscle fibre types are well established (Schiaffino & Reggiani, 2011), specific molecular signatures of MN type have been restricted to mice, usually at very young ages (Chakkalakal et al., 2010; Enjin et al., 2010; Kaplan et al., 2014), with many studies ignoring the physiologically relevant property of MN size (Dukkipati et al., 2018; Enjin et al., 2010; Fogarty, 2018). This is a major limitation in ageing research, as larger hypoglossal and lumbar MNs and type FF motor units appear more vulnerable to dysfunction, degeneration and death compared to type S and FR units (Cheung & Fogarty, 2026; Larsson et al., 2019).

In studies using murine models of amyotrophic lateral sclerosis (ALS), proposed molecular markers for type FF (and some FR) MNs, such as matrix metalloproteinase 9 (MMP‐9) and chondrolectin (Chodl) are lost with disease progression (Allodi et al., 2021; Dukkipati et al., 2018; Kaplan et al., 2014; Mei et al., 2025), consistent with the loss of larger MNs in human ALS *post mortem* tissue (Kiernan & Hudson, 1991). Despite preliminary indications that MMP‐9 and Chodl expression is restricted to type FF spinal MNs (Dukkipati et al., 2018; Enjin et al., 2010; Martinez‐Silva et al., 2018), there has been no substantive evaluation of hypoglossal MNs, which die in ageing and ALS (Fogarty, 2023; Kiernan & Hudson, 1991). Whether these molecular signatures are predominantly expressed in larger MNs when evaluated in the orientation providing the largest range of MN somal areas, transverse for hypoglossal (Altschuler et al., 1991) and parasagittal for spinal MNs (Sterling & Kuypers, 1976), is unclear. Thirdly, these molecular signatures have not been evaluated at older ages where neurodegenerative diseases are more common, or in old age where MN death may naturally occur.

In the ageing neuromotor system, deteriorations in motor control and coordination, the eventual weakness of type IIx/b fibres and impairments in maximal power‐requiring behaviours is evident. These changes indicate a selective vulnerability of type FF motor units (Fogarty & Sieck, 2019; Larsson et al., 2019). Similar pathophysiological patterns are observed in ALS and may relate to a shared aetiology of MN death (Fogarty et al., 2026; Pandya & Patani, 2020). In elderly humans, MN loss is relatively well‐established, with some indications of disproportionate death in larger (likely type FF) MNs (Cheung & Fogarty, 2026). These findings are mirrored in the hypoglossal and lumbar MNs of ageing Fischer 344 (F344) rats (Cheung & Fogarty, 2026). As MN somas are markedly plastic in response to stressors (Krishnan, 1983), efforts to evaluate somal size‐dependant MN death in ageing may be confounded. Our goal was to validate the size‐dependence (lower compared to upper tertile MN somal sizes), in the correct orientations, of MMP‐9 and Chodl in rat MNs and investigate whether FF type‐dependent vulnerability occurred in old hypoglossal and lumbar MNs.

In this study, we used quantitative immunohistochemical methods to evaluate MMP‐9 and Chodl in hypoglossal and lumbar MNs for the first time in rats. We tested three major hypotheses: (i) that MMP‐9 and Chodl would be predominantly expressed in larger hypoglossal and lumbar MNs in young rats; (ii) that MMP‐9 or Chodl will be a sensitive and specific predictor for larger somal size young rats; and (iii) in old age, the abundance of larger MMP‐9 and Chodl positive MNs will be reduced.

## 2 METHODS

### 2.1 Ethical approval

Animal use was performed in accordance with journal policy and approved by Mayo Clinic IACUC (no. A00008010‐24).

### 2.2 Experimental animals

Pathogen‐free F344 rats obtained from the National Institute on Aging and acclimated for at least 1 week before experiments. Age selection was based on F344 survival curves (Turturro et al., 1999) with young (6 months old; *n* = 10, 5 per sex) and old rats (24 months old; *n* = 10, 5 per sex) having 100% and 50% survival, respectively. Animals were maintained two per cage under an alternating 12:12 h light–dark cycle with ad libitum access to rat chow and fresh water. Experiments were performed between 08.00 and 11.00 h in the light phase.

### 2.3 Tissue collection

Rats were deeply anaesthetized with intraperitoneal injection of ketamine (80 mg/kg) and xylazine (20 mg/kg), euthanized by exsanguination, perfused with heparinized saline, then perfused with 4% paraformaldehyde (PFA) in 0.1 M phosphate‐buffered saline (PBS, pH 7.4). The brainstem and lumbar spinal cord was excised, post‐fixed in 4% PFA overnight and then immersed overnight in 25% sucrose in PBS.

### 2.4 Immunolabelling and imaging

Fixed tissue was subsequently embedded in cryomolds (VWR, Radnor, PA, USA), sectioned 40 µm (transverse for brainstem, parasagittal for spinal cord) and stored in 0.1 M PBS solution in 24‐well plates. Tissue sections were incubated for 72 h with the appropriate primary antibodies in 5% normal donkey serum in 0.2% Triton X‐100 in 0.1 M PBS. An anti‐MMP‐9 antibody (rabbit monoclonal, ab76003; abcam, Cambridge, UK; 1:300) and an anti‐Chodl antibody (human polyclonal, HPA057559; Atlas Antibodies, Stockholm, Sweden; 1:300) were used. An anti‐choline acetyltransferase (ChAT) antibody (goat polyclonal, AB144P; Sigma‐Aldrich, St Louis, MO, USA; 1:300) was used to label MNs. After being washed three times in 0.1% Triton X‐100 in 0.1 M PBS (10 min each), sections were incubated with donkey Alexa Fluor 555 anti‐rabbit (cat. no. 711‐545‐152) and Alexa Fluor 647 anti‐goat (cat. no. 705‐605‐147) or Alexa Fluor 555 anti‐goat (cat. no. 705‐565‐147) and Alexa Fluor 647 anti‐rabbit (cat. no. 711‐606‐152; Jackson Immuno Research Laboratories, Inc., West Grove, PA, USA) secondary antibodies for 3 h. Tissue sections were then washed three times 0.1% Triton X‐100 in 0.1 M PBS (10 min each) before being mounted with Vectashield Antifade Mounting Media (cat. no. H‐1900; Vector Laboratories Inc., Newark, CA, USA) and cover‐slipped.

ChAT‐labelled MNs were visualized using a Nikon A1R Confocal Laser Scanning Microscope (RRID:SCR_020317) equipped with argon (488 nm), green HeNe (543 nm) and red HeNe (633 nm) lasers. All images were acquired at 12‐bit resolution in an array of 1024 × 1024 pixels using a ×60 (NA 1.40) oil immersion objective lens with a step size of 2 µm, consistent with prior detailed 3D MN imaging (Fogarty et al., 2023). Sequential imaging was performed using a dichroic mirror beam splitter that allows transmission of 555–615 nm (reflects 480–555 and 615–800 nm) and appropriate band‐pass emission filters (495–535 nm and 575–630 nm long pass for Alexa 488 and 594, respectively) to reduce cross‐talk (Cheung, Bhavnani et al., 2024; Cheung, Willis et al., 2024; Fogarty et al., 2013). Laser intensity and photomultiplier settings were adjusted to less than 10% of the dynamic range when imaging negative control sections to ensure the background was true black and no pixels were overexposed. Imaging parameters were kept consistent between all sections of each rat to allow for intensity quantification.

### 2.5 Quantifying MMP‐9 and Chodl

Regions of interest (ROIs) were manually drawn around each ChAT‐positive lumbar and hypoglossal MN using ImageJ to measure MMP‐9 and Chodl intensity. To ensure consistent intensity measurements, only MNs with a visible nucleus within the middle 25% of the 40 µm z‐stack were sampled. To prevent lipofuscin autofluorescence from confounding intensity measurements, autofluorescent puncta in the 488 nm channel (comprising brightest autofluorescence) was threshold using ImageJ's ‘Minimum’ algorithm following Auto Brightness/Contrast and subtracted from the MN ROI, to measure only MMP‐9 and Chodl intensity at 555 and 647 nm, respectively. A 10 × 10 µm ROI was used to measure background intensity adjacent to each analysed MN in the 555 and 647 nm channels, then subtracted from the MMP‐9 and Chodl signal to calculate the true MN MMP‐9 and Chodl intensity. To allow comparison across rats, the presented MMP‐9 and Chodl intensities were normalized within each animal to a 0–100 scale. MNs with normalized intensities greater than 50 were considered MMP‐9‐ and Chodl‐positive for paired analysis.

### 2.6 Determining MN sizes and somal surface area tertiles

The *x* and *y* radii of every hypoglossal and lumbar MN was measured using ImageJ, with surface areas calculated using the prolate spheroid approximation (Ulfhake & Cullheim, 1988). For tertile stratification, the mean of each lower and upper tertile boundary for young rats is calculated, and all MNs across rats of all ages separated according to where they lay across these boundaries (Fogarty et al., 2018). Lumbar MNs with an average *x* and *y* radius of <25 µm and hypoglossal MNs of <15 µm were excluded.

### 2.7 Statistical analysis

We used Prism 10 for all data analyses (GraphPad Software, Boston, MA, USA). Each data set was assessed for normality with a D'Agostino and Pearson test. For multiple comparisons (age and size tertile), two‐way paired ANOVA with Fisher's LSD test was performed. *xy* plots were analysed using linear regression. All data are reported as the mean ± standard deviation, unless otherwise specified, and outliers, determined a priori as values ≥2.5 standard deviations from the group mean, were excluded from analyses. Statistical significance was established at the *P *< 0.05 level. For the major outcomes, Cohen's *d* was used to allow for reliable and robust comparisons between groups. Analyses were performed blind to age, size of MN and sex.

## 3 RESULTS

### 3.1 MMP‐9 expression in lumbar MNs of young and old rats

MMP‐9 expression was observed in small and large lumbar MNs in young and old rats (Figure 1). When somal surface areas of lumbar MNs were divided based on the 6‐month population tertile, there was a modest but significant positive association between somal surface area and MMP‐9 intensity in 6‐month‐old (*P* = 0.0001, *R* ^2^ = 0.050; Figure 1c), but not 24‐month‐old rats (*P* = 0.858, *R* ^2^ = 0.000; Figure 1d). Paired ANOVA analysis revealed that the percentage of MMP‐9 positive (>50% of normalized MMP‐9 intensity) MNs in young rats was approximately double (*P* = 0.004, Cohen's *d* = 2.36) in the larger tertile, whereas no difference was observed between tertiles in old rats (*P* = 0.85; Figure 1e).

**FIGURE 1:**
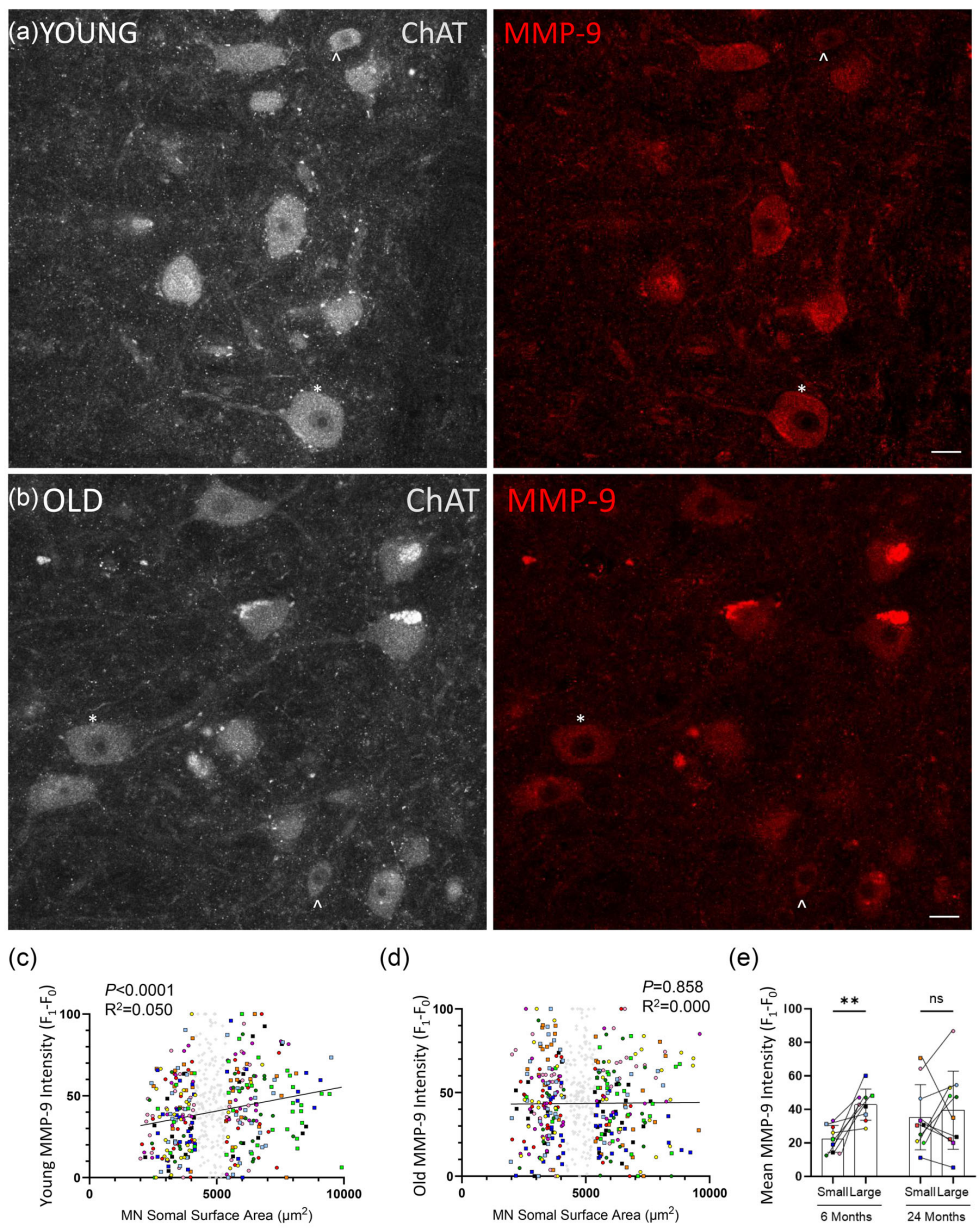
(a) MMP‐9 in small (^) and large (*) lumbar MNs in 6‐month‐old rats. (b) MMP‐9 in small (^) and large (*) lumbar MNs in 24‐month‐old rats. (c) *xy* plots of young rat MMP‐9 intensity versus somal surface area for small and large tertile lumbar MNs in old rats (*n* = 312 MNs). (d) *xy* plots of old rat MMP‐9 intensity versus somal surface area for small and large tertile lumbar MNs in old rats (*n* = 334 MNs). Different colours represent small and large tertile values for each rat. Grey represents middle tertile. (e) Paired two‐way ANOVA for the mean percentage of MMP‐9‐positive lumbar MNs between small and large size tertile in young (*n* = 9 rats, 1 excluded due to mean value >2.5 times the group SD [see Methods]) and old rats (*n* = 10 rats). ns, *P* = 0.85; **P = 0.004.

The size distributions of MMP‐9 positive and negative lumbar MNs was assessed in young and old rats. We observed a difference between MMP‐9 positive and negative MN size‐distributions in young rats (*P* = 0.033, Kolmogorov–Smirnov test; Figure 2a), but not old (*P* = 0.136, Kolmogorov–Smirnov test; Figure 2b).

**FIGURE 2:**
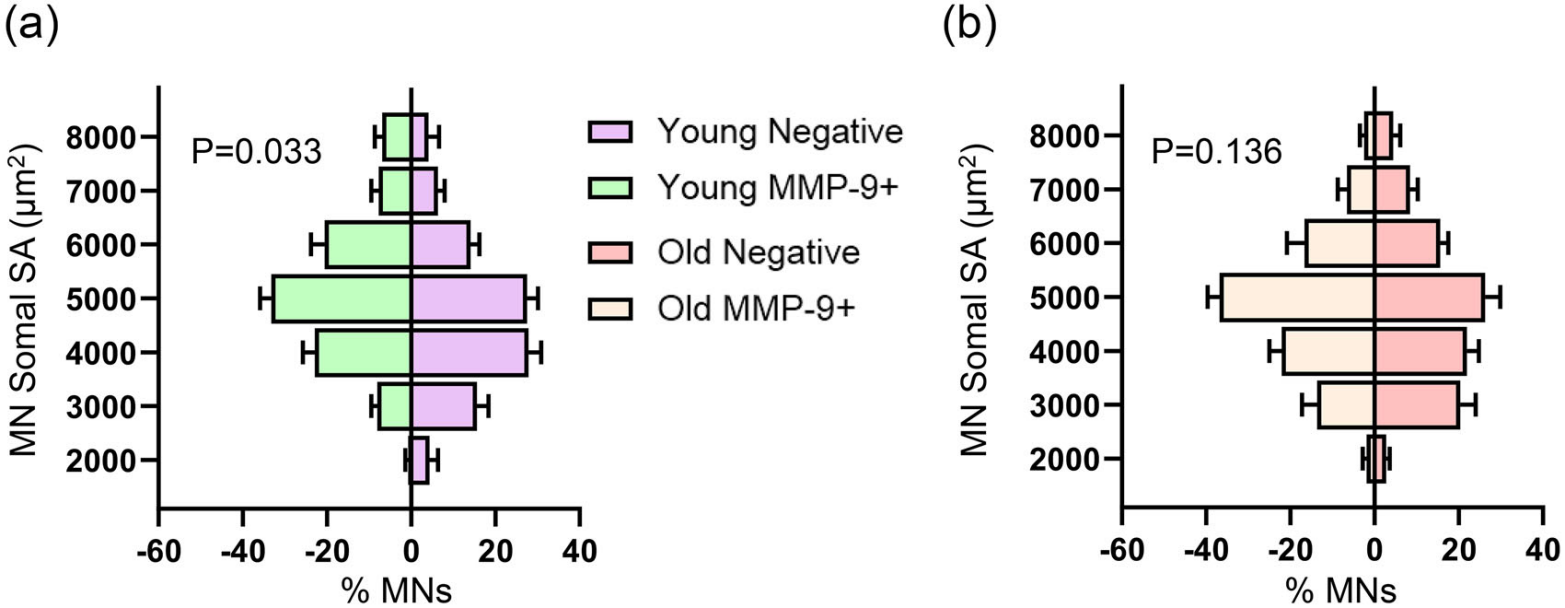
(a) In young rats, frequency histogram of MN somal size differs between MMP‐9 positive and negative neurons (Kolmogorov–Smirnov test, *P* = 0.033). (b) In old rats, frequency histogram of MN somal size is unchanged between MMP‐9 positive and negative neurons (Kolmogorov–Smirnov test, *P* = 0.136).

### 3.2 Chodl expression in lumbar MNs of young rats

Low Chodl expression was observed in 6‐month‐old lumbar MNs (Figure 3a). Somal surface area showed no significant association with Chodl expression (*P* = 0.480, *R* ^2^ = 0.002; Figure 3b). Paired analysis revealed no difference in mean Chodl positive percentage between small and large lumbar MNs (*P *> 0.05; Figure 3c).

**FIGURE 3:**
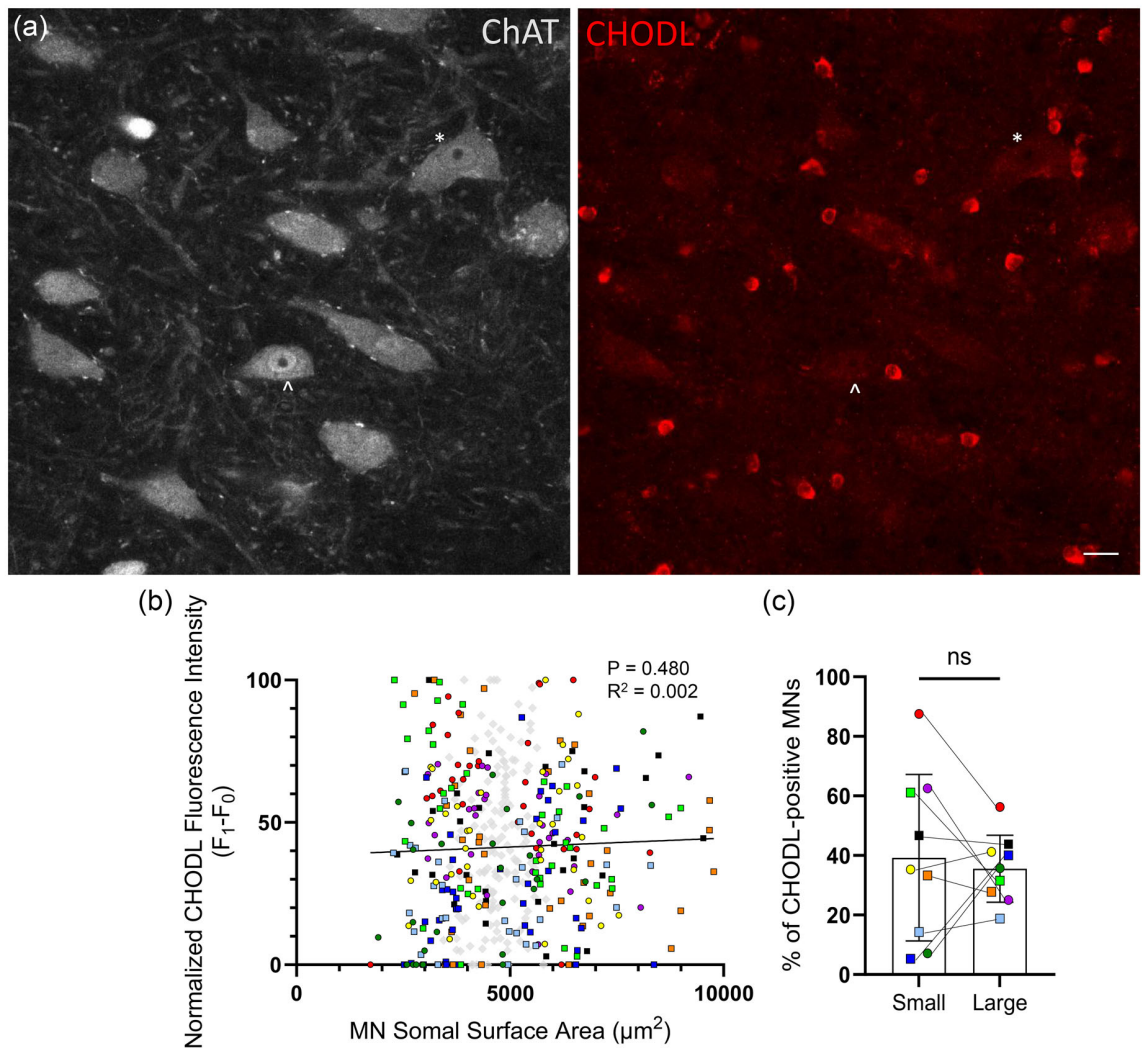
(a) Chodl in small (^) and large (*) lumbar MNs. (b) *xy* plots show no size‐dependence of Chodl intensity (*n* = 299 lumbar MNs). Grey represents middle tertile. (c) Paired Student's *t*‐test showed unchanged mean percentage of Chodl positive MNs between small and large lumbar MN size tertiles (*n* = 10 rats).

### 3.3 MMP‐9 and Chodl expression in hypoglossal MNs of young rats

Low MMP‐9 expression was observed in 6‐month‐old hypoglossal MNs (Figure 4a). Paired analysis revealed no difference in the mean percentage of MMP‐9 positive hypoglossal MNs between small and large size (*P *> 0.99; Figure 4c). Hypoglossal MN somal surface area showed no significant association with MMP‐9 expression (*P* = 0.269, *R* ^2^ = 0.004; Figure 4e).

**FIGURE 4:**
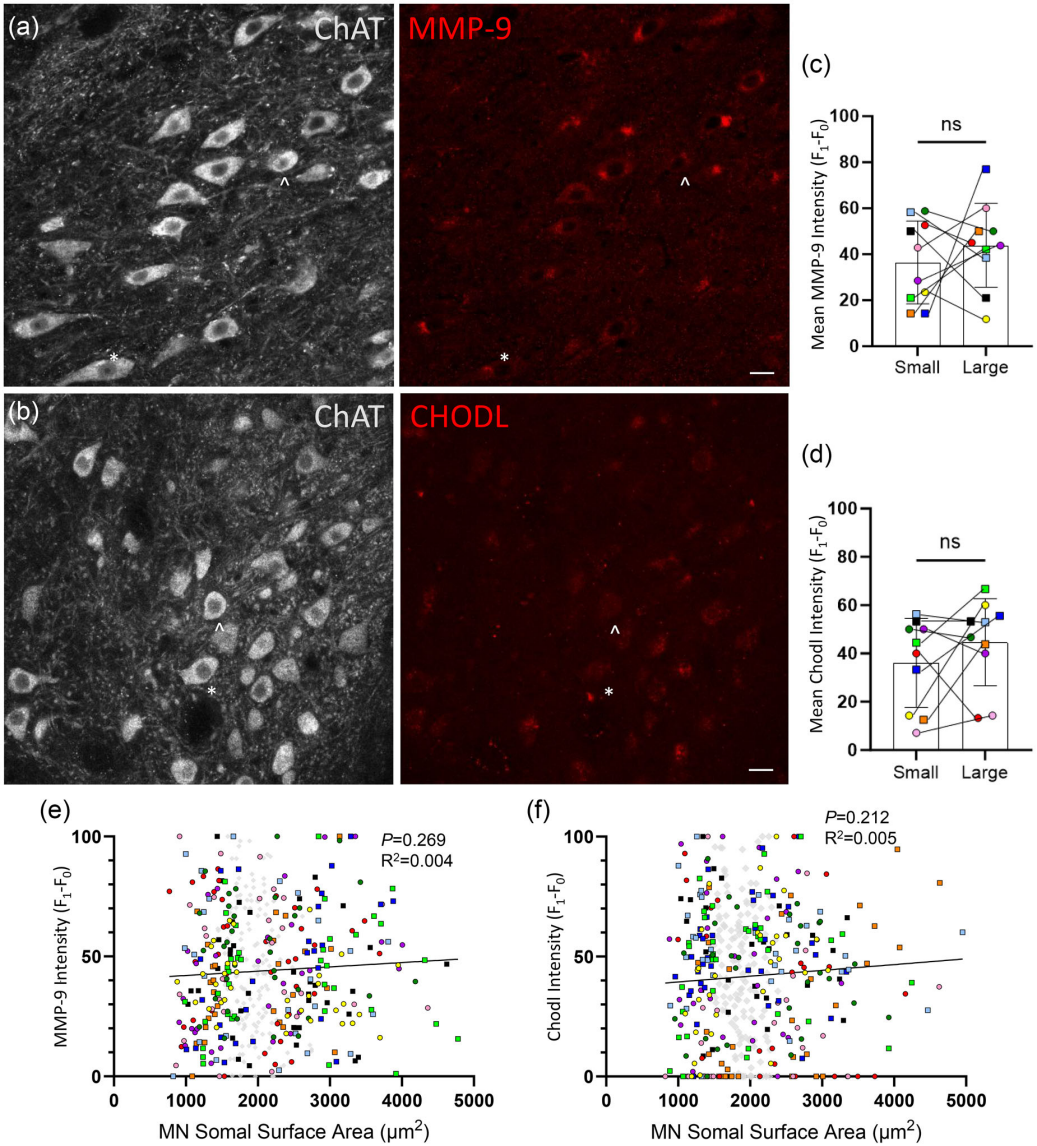
(a) MMP‐9 in small (^) and large (*) hypoglossal MNs. (b) Chodl in small (^) and large (*) hypoglossal MNs. (c) Paired Student's *t*‐test showed unchanged mean percentage of MMP‐9 positive MNs between small and large hypoglossal MN size tertiles (*n* = 10 rats). (c) Paired *t*‐test showed unchanged mean percentage of Chodl positive MNs between small and large hypoglossal MN size tertiles (*n* = 10 rats). (e) *xy* plots show no size‐dependence of MMP‐9 intensity (*n* = 325 hypoglossal MNs). (f) *xy* plots show no size‐dependence of Chodl intensity (*n* = 325 hypoglossal MNs). Different colours represent small and large tertile values for each rat. Grey represents middle tertile.

Low Chodl expression was observed in 6‐month‐old hypoglossal MNs (Figure 4b). Paired analysis revealed no difference in the mean percentage of Chodl positive hypoglossal MNs between small and large size (*P* = 0.79; Figure 4d). Hypoglossal MN somal surface area showed no significant association with MMP‐9 expression (*P* = 0.212, *R* ^2^ = 0.005; Figure 4f).

## 4 DISCUSSION

Age‐related brainstem and spinal cord MN loss is a key component of diminished neuromotor function and weakness in ageing, which largely affects muscle fibres and behaviours involving type FF motor units (Fogarty & Sieck, 2019; Larsson et al., 2019). Here, we present three major advances in motor unit neurobiology. Firstly, we found that MMP‐9 is a moderate sensitive and specific predictor of large MNs in a rat lumbar sample cut parasagittally. Second, the proportion of large MMP9 positive MNs declines with old age in the lumbar MNs, consistent with a loss of type FF motor units. Third, we found that in hypoglossal MNs neither MMP9 nor Chodl had differential expression between smaller and large MNs, with chodl also failing to be expressed in a size‐dependent manner in lumbar MNs. These results are of great importance to the study of motor units specific MN neurobiology in adult rats.

In F344 rats, age‐related hypoglossal MN death and neuromuscular junction denervation of tongue muscles is consistent with tongue weakness and deficits in aerodigestive behaviours (Christensen & Fogarty, 2026; Fogarty, 2025; Fogarty et al., 2026; Kemp et al., 2026; Sieck et al., 2023). A modest size‐dependence has been observed in old age brainstem MN loss, although not in all regions (Fogarty, 2023; Fogarty, 2025; Fogarty et al., 2026), with death of MNs occurring precipitously from late‐middle to old age. Here, the expression of MMP‐9 and Chodl in hypoglossal MNs was not consistent with the size principle, despite the abundance tongue muscle fibres expressing type IIx/b MyHCs. The lack of size‐dependent MMP‐9 or Chodl expression in hypoglossal MNs may reflect intrinsic molecular differences between brainstem and spinal MNs. Notably, hypoglossal MNs arise from distinct hindbrain developmental lineages compared to lumbar MNs (Philippidou & Dasen, 2013). These developmental differences may result in diverse molecular regulation of MMP‐9 expression. Obviously, much more needs to be determined regarding the molecular profiling of brainstem MNs, particularly regarding vulnerable and resilient motor unit types in ALS and ageing.

In spinal cord MNs and limb muscles, the selective vulnerability of type FF motor units is highly characterized in type IIx/b fibres from limb muscles of humans and rodents (Larsson et al., 2019; Rooker et al., 2026). Larger lumbar MNs showed positive association with MMP‐9 expression, and a greater percentage of MMP‐9 positive MNs compared to small, consistent with the size principle. In old rats, the size‐dependant and tertile relationship was lost. Our findings raise the possibility that this MMP‐9 positive subpopulation is preferentially vulnerable during ageing. Alternatively, as MMP‐9 participates in a positive feedback loop with oxidative stress (Kar et al., 2010) and chronic inflammation, which increase with ageing (Fogarty et al., 2026; Katharesan et al., 2016), surviving large MNs may downregulate their MMP‐9 expression as an adaptive response to limit oxidative damage. These mechanisms are not mutually exclusive and future studies are required to determine whether the loss of tertile‐specific MMP‐9 expression in ageing reflects selective degeneration, adaptive downregulation or a combination of both processes.

Discrepancies between our results and those reported previously may be due to several factors. First, the majority of previous studies investigating MMP‐9 and Chodl were performed in C57BL/6 mice. Many of these murine studies (and almost all Chodl investigations) dealt exclusively with mRNA, not protein. Second, previous studies investigated transverse sections of the lumbar spinal cord, whereas we evaluated in the orientation providing the largest range of measurable MN somal areas, transversely for the brainstem hypoglossal MNs (Altschuler et al., 1991; Kanjhan et al., 2016) and parasagittally oriented for spinal lumbar MNs (Sterling & Kuypers, 1976). Thirdly, MMP‐9 has primarily been identified as a marker of selectively vulnerable MNs in ALS (Kaplan et al., 2014) and may be related to the many physiological roles of MMP‐9. Indeed, within MNs MMP‐9 is proposed to act cell‐autonomously to enhance endoplasmic reticulum stress (Kaplan et al., 2014). Additionally, MMP‐9 may promote inflammatory death pathways (Kiaei et al., 2007), synaptic plasticity (Legutko et al., 2025) and degrades the neuroprotective extracellular matrix (Cheung, Bhavnani et al., 2024; Cheung, Willis et al., 2024). Chodl expression in ‘fast’ MNs was assessed in 11‐day‐old mice (Enjin et al., 2010), before the onset of mature motor unit types and MN properties (Fogarty & Sieck, 2019), perhaps indicating it plays more of a role in maturation rather than identification.

Overall, MMP‐9 was a modest indicator of MN size in young lumbar MNs. In old age, homogeneous MMP‐9 expression in lumbar MNs across size was suggestive of alterations within vulnerable MN populations. Differences in MMP‐9 expression patterns between hypoglossal and lumbar MNs suggest that large MNs may comprise multiple molecularly distinct subpopulations, complicated by the multiple physiological roles of MMP‐9. Our findings provide new insights into the molecular basis of selective MN vulnerability during ageing and establish a foundation for future studies aimed at preserving motor units in age‐related motor dysfunction.

## AUTHOR CONTRIBUTIONS

Matthew J. Fogarty conceived and designed project. Sang Won Cheung and Matthew J. Fogarty performed experiments. Sang Won Cheung and Matthew J. Fogarty analysed data. Sang Won Cheung and Matthew J. Fogarty drafted and revised the manuscript and figures. All authors approved the final version of the manuscript and agree to be accountable for all aspects of the work, ensuring that questions related to the accuracy or integrity of any part of the work are appropriately investigated and resolved. All persons designated as authors qualify for authorship, and all who qualify for authorship are listed.

## CONFLICT OF INTEREST

None declared.

## GENERATIVE AI STATEMENT

No generative AI was used in the production of this work.

## Data Availability

Data are available from the corresponding author by request.
